# Follow-Up Investigation on the Promotional Practices of Electric Scooter Companies: Content Analysis of Posts on Instagram and Twitter

**DOI:** 10.2196/16833

**Published:** 2020-01-23

**Authors:** Allison Dormanesh, Anuja Majmundar, Jon-Patrick Allem

**Affiliations:** 1 Keck School of Medicine of USC Los Angeles, CA United States

**Keywords:** electric scooter, scooters, public safety, road safety, social media, marketing, technology, ride sharing, public health

## Abstract

**Background:**

Electric scooters (e-scooters) have become a popular mode of transportation in both the United States and Europe. In the wake of this popularity, e-scooters have changed the commuting experience in many metropolitan areas. Although e-scooters offer an efficient and economical way to travel short distances in traffic-congested areas, recent studies have raised concerns over their safety. Bird and Tier Mobility are 2 popular e-scooter companies in the United States and Europe, respectively. Both companies maintain active social media accounts with hundreds of posts and tens of thousands of followers. Recent studies have shown that consumer behavior may be influenced by the content posted to popular social media platforms, such as Instagram and Twitter.

**Objective:**

This study aimed to examine the official Instagram and Twitter accounts of Bird and Tier Mobility to determine whether these companies promote and demonstrate the use of safety gear in their posts to their consumers.

**Methods:**

Posts to Bird’s (n=287) and Tier Mobility’s (n=190) official Instagram accounts, as well as Bird’s (n=313) and Tier Mobility’s (n=67) official Twitter accounts, were collected from November 9, 2018, to October 7, 2019. Rules for coding content of posts were informed by previous research.

**Results:**

Among posts to Bird’s Instagram account, 69.3% (199/287) had a person visible with an e-scooter, 9.1% (26/287) contained persons wearing protective gear, and there were no mentions of protective gear in captions corresponding to the post. Among posts to Tier Mobility’s Instagram account, 84.7% (161/190) contained a person visible with an e-scooter, 36.3% (69/190) contained persons wearing protective gear, and 4.2% (8/190) of captions corresponding to posts mentioned protective gear. Among posts to Bird’s Twitter account, 71.9% (225/313) had an image, of which 44.0% (99/225) contained a person visible with an e-scooter and 15.1% (34/225) contained persons wearing protective gear. Among posts to Tier Mobility’s Twitter account, 78% (52/67) had an image, of which 52% (27/52) contained a person with an e-scooter and 21% (11/52) contained persons wearing protective gear.

**Conclusions:**

Findings show that modeling and promoting safety is rare on Bird’s and Tier Mobility’s official social media accounts, which may contribute to the normalization of unsafe riding practices. Social media platforms may offer a potential avenue for public health officials to intervene with rider safety campaigns for public education.

## Introduction

### Background

Over the past 3 years, electric scooters (e-scooters) have become a popular mode of transportation in both the United States and Europe [1]. In the wake of this popularity, the commuting experience in many metropolitan areas has changed. E-scooter companies offer rentable, dockless, generally affordable, single-rider e-scooters that can reach speeds of 15 miles per hour and traverse 30 miles on 1 charge. Consumers can access these e-scooters through Web or downloadable apps on their smartphones. Once logged in, each user can be directed to the closest e-scooter available via global positioning system. After finishing their ride, users can leave the e-scooter anywhere within the legal parking zones, indicated on their mobile app.

Although e-scooters offer an efficient and economical way to travel short distances in traffic-congested areas [1,2], recent studies have raised concerns over their safety. For instance, the Austin Public Health Department and Center for Disease Control and Prevention recently published a study on injuries and risk factors associated with rentable, dockless e-scooter use [3]. They found that often the injuries sustained were to the rider’s head (48%), and only 1 in 190 riders were wearing a helmet [3]. A second study on e-scooter injuries from Southern California recorded 249 injuries associated with e-scooter use over the course of 1 year [4]. It found that 80% of riders were injured from a fall, and 10 out of 249 riders were wearing a helmet (only 4% of all riders) [4]. European countries like Germany have also experienced a rise in the use of e-scooters, and subsequent reports of injuries. Within a 3-month period, police statistics showed that 74 e-scooter-related accidents occurred in Berlin, and over 200 e-scooter riders were cited for traffic violations [5]. Taken all together, the growing number of injuries from the use of e-scooters highlights the importance of promoting safe riding practices among e-scooter customers.

Previous research has suggested that the promotional activities from companies on popular social media platforms may influence consumer behavior [5], and this influence may extend to the perceived norms relating to e-scooter safety, for example, wearing safety gear [6]. In a previous study analyzing data from 2017 to 2018, Allem and Majmundar demonstrated that posts to Bird’s (one of the leading US e-scooter companies) official Instagram account rarely (6% of the time) showed users wearing protective gear when photographed with an e-scooter [6]. Although this study was the first to characterize any social media activity from an e-scooter company, additional research is needed that considers multiple social media platforms from multiple e-scooter companies to better understand promotional practices pertaining to e-scooter safety.

The e-scooter industry is characterized by high growth [4] and intense competition inside and outside of the United States [1]. Bird, for example, is valued at over US $2 billion [2,7], and as of October 2019, has been funded US $275 million to expand further [8]. Similarly, European countries are experiencing demand for e-scooter options to lower traffic congestion, increase parking availability, and improve air quality [1]. For instance, as of May 2019, Germany legalized e-scooters [9], and as a result, Tier Mobility (a Berlin-based start-up company) has made efforts to change Germany’s public transportation infrastructure, including partnerships with public transport, municipal service, and private mobility providers [10]. Their main goal is to use these partnerships to change the status quo of urban mobility [10]. Tier Mobility, which started about a year after Bird in 2018, has quickly received 10 million rides in the 11 months since they have launched, a majority of which occurred between June and October of 2019 [11]. This user activity is comparable with Bird’s user activity in the United States (Bird achieved 10 million rides in its first year) [12]. Although other companies, for example Uber, provide e-scooters as modes of transportation in the United States and abroad, Uber offers and promotes bicycles and other ridesharing services. Bicycles are distinct from e-scooters—subjected to different laws and risks from riding on the road. As a result, this study examined the safety promotions of companies (Bird and Tier Mobility) that prioritize e-scooters over other ridesharing options.

The promotion of safe riding practices on behalf of e-scooter companies is ever pressing, especially on popular social media platforms. In 2018, Bird went on record stating that it utilizes, “targeted advertising with safety messages on social media platforms” [13]. Tier Mobility has also been on record encouraging its consumers to wear helmets, as well as engage with their introductory tutorial to learn how to avoid accidents [14].

### Objective

This study revisits the promotional practices of Bird on Instagram to include their most recent posts, and goes beyond previous research by including the Instagram posts from a second comparable company, Tier Mobility. In addition, this study includes Twitter posts from Bird and Tier Mobility. Findings could inform health communication campaigns aimed at promoting safe e-scooter practices.

## Methods

Posts, including images and captions, were collected from Bird’s (n=287) and Tier Mobility’s (n=190) official Instagram accounts and Bird’s (n=313) and Tier Mobility’s (n=67) official Twitter accounts between November 9, 2018, and October 7, 2019.

### Instagram Analysis

Similar to previous research [6], each Instagram post was reviewed by 1 author and characterized as to whether (1) person(s) was or were visible in the post with an e-scooter, (2) person(s) in the post was or were wearing any protective gear (eg, if any of the following were present on the person(s): helmet, wrist guards, elbow pads, or knee pads, then protective gear was coded as present), (3) protective gear was visible anywhere in the post, (4) protective gear or safety was mentioned in the captions corresponding to the post, (5) the post was a *repost* or the photo credited to a customer of the company and adopted for their own use, and (6) number of likes. To establish interreliability, a second investigator coded a subsample of posts (n=50) from Bird’s Instagram account. Agreement ranged from 92% to 100% for the coded categories. The number of followers from each Instagram account were also recorded.

### Twitter Analysis

Each Twitter post was reviewed by 1 author and characterized as to whether (1) it contained an image (eg, pictures and videos), (2) person(s) was or were visible in the post with an e-scooter, (3) person(s) in the post was or were wearing any protective gear (eg, if any of the following were present on the person(s): helmet, wrist guards, elbow pads, or knee pads, then protective gear was coded as present), (4) protective gear was visible anywhere in the post, (5) protective gear or safety was mentioned in the post, (6) the number of likes, and (7) the number of *retweets*. To establish interreliability, a second investigator coded a subsample of posts (n=50) from Bird’s Twitter account. Agreement ranged from 90% to 100% for the coded categories. The number of followers from each Twitter account were also recorded.

All analyses relied on publicly available data, accessible through Instagram’s or Twitter’s website or mobile device app. This study adhered to the terms and conditions, terms of use, and privacy policy of Instagram and Twitter. Descriptive statistics were reported for each category.

## Results

### Analysis of Instagram Posts

The Instagram accounts of Bird and Tier Mobility had approximately 89,000 followers and 12,000 followers, respectively. Among posts to Bird’s Instagram account, 69.3% (199/287) had a person visible with an e-scooter, 9.1% (26/287) contained persons wearing protective gear, 11.5% (33/287) contained protective gear somewhere in the post, and there were no mentions of protective gear in the captions (see Multimedia Appendix 1 for example posts). About 53.3% (153/287) of Bird’s posts were reposts, and among reposts, 2.9% (4/153) had persons wearing protective gear. Likes per post ranged from 89 to 28,926 (mean 1155.69, median 598).

Among posts to Tier Mobility’s Instagram account, 84.7% (161/190) contained a person visible with an e-scooter, 36.3% (69/190) contained persons wearing protective gear, 44.7% (85/190) had protective gear somewhere in the post, and 4.2% (8/190) of captions corresponding to the post mentioned safety. About 8.9% (17/190) of Tier’s posts were reposts, and among reposts, 2% (1/17) had persons wearing protective gear. Likes per post ranged from 34 to 5190 (mean 367.88, median 139.50).

### Analysis of Tweets

The Twitter accounts of Bird and Tier Mobility had about 17,000 followers and 2000 followers, respectively. Among Bird’s Twitter posts, 71.9% (225/313) had an image, of which 44.0% (99/225) had a person visible with an e-scooter, 15.1% (34/225) contained persons wearing protective gear, and 21.3% (48/225) contained protective gear somewhere in the post. Among all posts, 5.8% (18/313) mentioned safety. Likes per post ranged from 0 to 398 (mean 25.58, median 16.00), whereas retweets per post ranged from 0 to 83 (mean 4.57, median 2.00). Among Tier Mobility’s Twitter posts, 78% (52/67) had an image, of which 52% (27/52) contained a person visible with an e-scooter, 21% (11/52) contained persons wearing protective gear, and 25% (13/52) had protective gear somewhere in the post. Among all posts, 5% (3/67) mentioned safety. Likes per post ranged from 1 to 54 (mean 12.04, median 8.00), whereas retweets ranged from 0 to 16 (mean 3.49, median 2.00).

## Discussion

### Overall Findings

Posts to the official social media accounts of Bird and Tier Mobility seldomly showed e-scooters being used with protective gear. In addition, findings showed that Bird and Tier Mobility utilize customers’ photos of their e-scooter experiences in promotions through reposts on Instagram. These reposts rarely showed e-scooters being used with safety in mind. Posts from both companies on both platforms received likes from their followers demonstrating engagement with the promotional content.

Findings from this study are similar to an earlier report demonstrating that posts to Bird’s Instagram account rarely showed riders wearing protective gear when photographed with e-scooters [6]. Findings from this study call into question Bird’s claims regarding their use of advertising with safety messages on social media platforms [13]. Taken all together, rider safety does not seem to be modeled or promoted on the social media accounts of popular e-scooter companies.

Although e-scooter companies state that they always encourage riders to wear a helmet [15], such companies could take advantage of the popularity of their social media accounts, and the impact of social media, by highlighting the advantages of using protective gear while riding e-scooters, and through other forms of education (eg, short videos). Educating consumers about the functional benefits of each type of protective gear can help reduce the impact of injuries from accidents (eg, kneepads can help prevent serious bruises in case of a fall and helmets can help prevent severe head injuries).

Bird’s primary avenue for providing users with safety suggestions is through their mobile phone app or website, which includes (1) suggestions of where to ride and park, (2) requiring users to be older than 18 years to ride, (3) allowing only 1 rider per vehicle, (4) providing traffic rules, and (5) suggesting helmet use [13]. Bird users can click on a safety tab, on the mobile app, and get directed to a page that allows them to order a helmet for US $9.99 (shipping cost). They also provide an optional *how to ride* safety tutorial. Similarly, Tier Mobility urges helmet use through their *how Tier works* tab on their website [16]. In contrast to Bird, Tier Mobility does not currently offer free helmets. Although both companies give indication of safety measures for their users, posts to social media platforms could further influence their users’ attitudes and behaviors [17]. The majority of e-scooter injuries are among those who are 18 to 29 years of age, an age group that often engages with social media [3]. In this study, engagement was measured by recording the number of *likes* and *retweets* received by Bird’s and Tier Mobility’s followers. Retweets represent approval for the content of the post [18] and can perpetuate such content throughout the Twittersphere [19].

Previous studies have highlighted the importance of safety gear as a preventive measure for rider injuries. Thompson et al analyzed 5 case-control studies and determined that helmets can reduce the risk of head, brain, and severe brain injury by 63% to 88% [20]. Although their study focused on bicyclists, protective gear could provide similar protection for other 2-wheel vehicles like e-scooters. Bird’s and Tier Mobility’s lack of promotion of safety gear on social media, in combination with a lack of warnings within post’s corresponding caption, may influence how riders understand the safety of these emerging modes of transportation.

### Limitations

Our findings are limited to posts on Instagram and Twitter and may not pertain to posts on other social media platforms like Snapchat. Our findings are also limited to 2 e-scooter companies (Bird and Tier Mobility) and may not pertain to other companies. Although companies like Lime, Lyft, and Uber offer numerous modes of transportation, including ridesharing cars, bicycles, and e-scooters, Bird and Tier Mobility focus on e-scooters. E-scooters have a specific set of risks from use, as well as specific laws to follow, and as a result, Bird and Tier Mobility were the focus of this study. The posts in this study were collected from an 11-month period and may not extend to other time periods. This study could not determine whether posts to Twitter or Instagram directly influenced consumer behavior and did not determine if protective gear would be effective in preventing injuries. However, previous research has shown that wearing helmets can mitigate the extent of injuries from motorized vehicles [20], and that communications on social media platforms may influence behavior [21].

### Conclusions

Findings from this study show that modeling and promoting safety is rare on Bird’s and Tier Mobility’s official social media accounts. These promotional practices may contribute to the perceived norms relating to e-scooter safety. Social media may offer a potential avenue for public health officials to intervene with promotional messages of their own to increase safe riding practices.

## Appendix

Multimedia Appendix 1 Example posts from Instagram and Twitter.

## Abbreviations

e-scooter: electric scooter
