# Supplementary material for: Follow-Up Investigation on the Promotional Practices of Electric Scooter Companies: Content Analysis of Posts on Instagram and Twitter
Source: JMIR Public Health Surveill. 2020 Jan 23;6(1):e16833. doi: 10.2196/16833 (PMC7005689; doi:10.2196/16833)
Supplement: Multimedia Appendix 1 [file publichealth_v6i1e16833_app1.docx]

Supplemental file

| Example post from Tier Mobility’s Instagram account with helmet |
| --- |
| 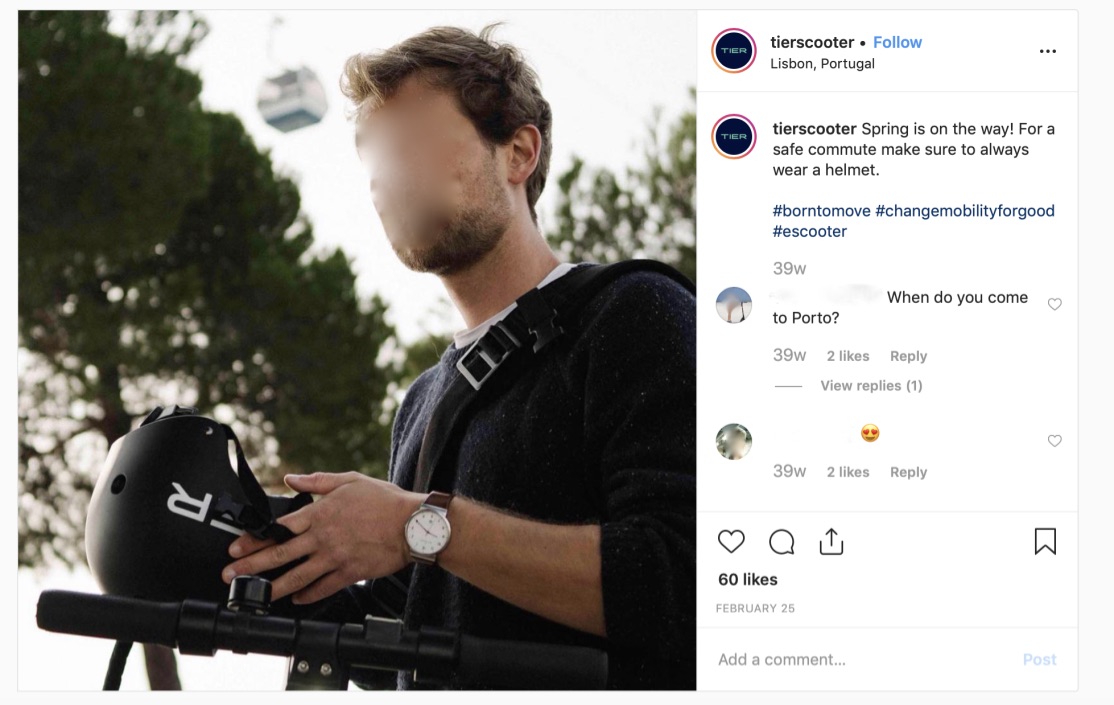 |

| Example post from Tier Mobility’s Instagram account without helmet |
| --- |
| 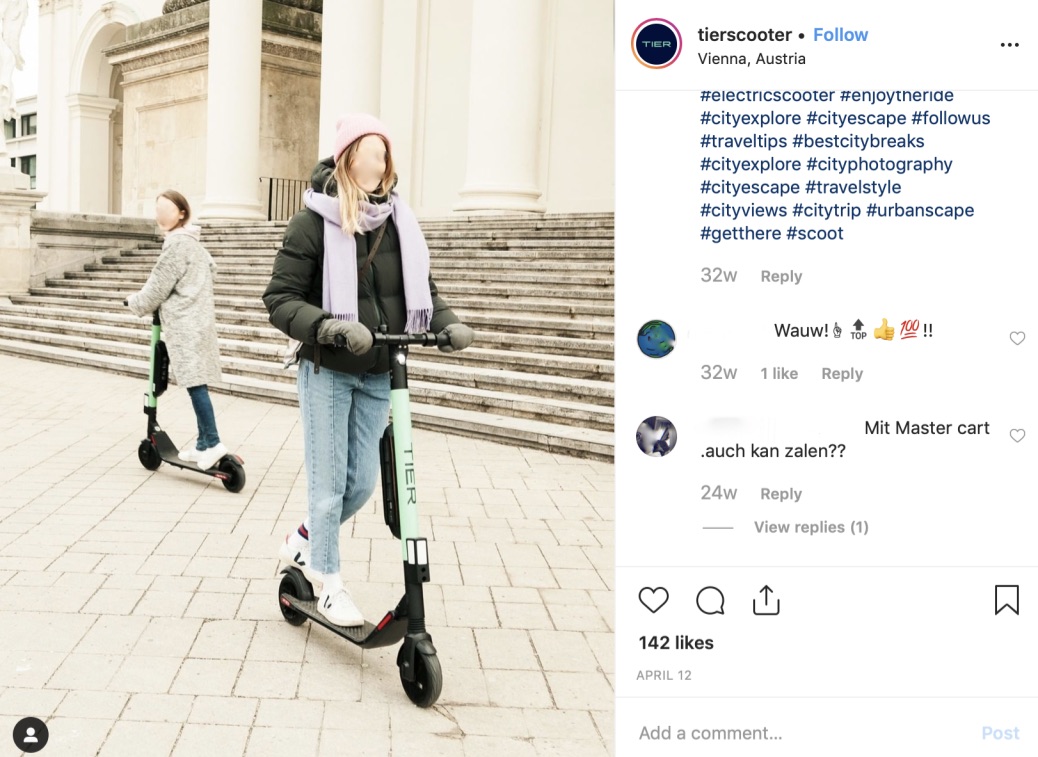 |

| Example post from Bird’s Twitter account without helmet |
| --- |
| 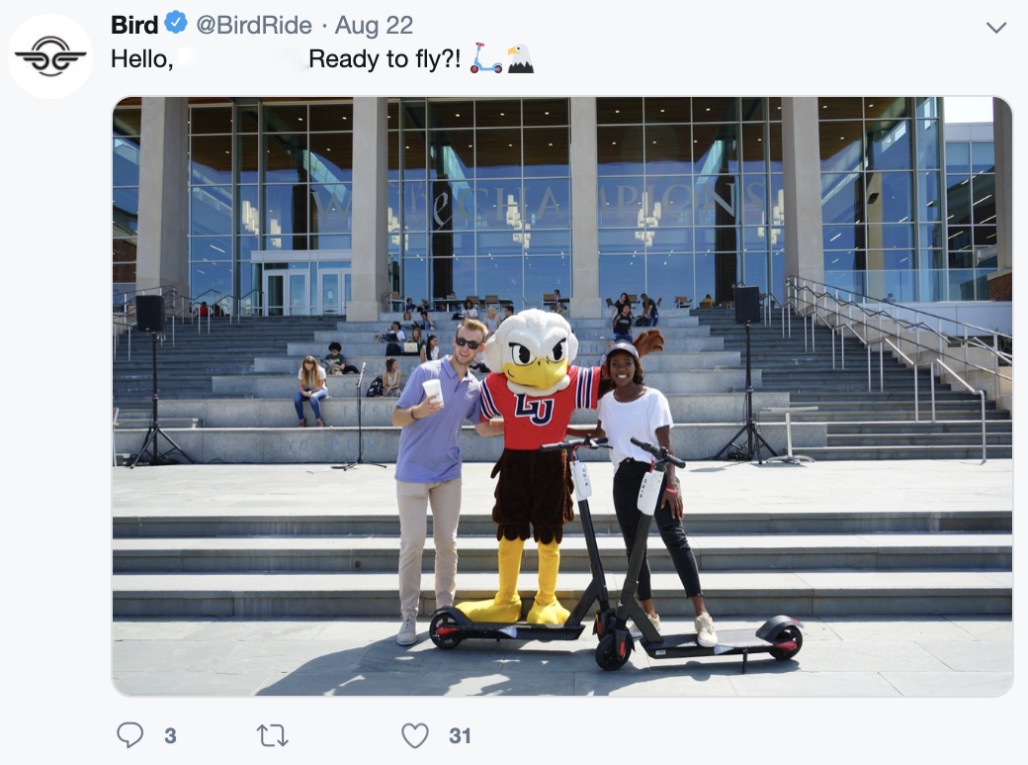 |

| Example post from Bird’s Twitter account with helmet |
| --- |
| 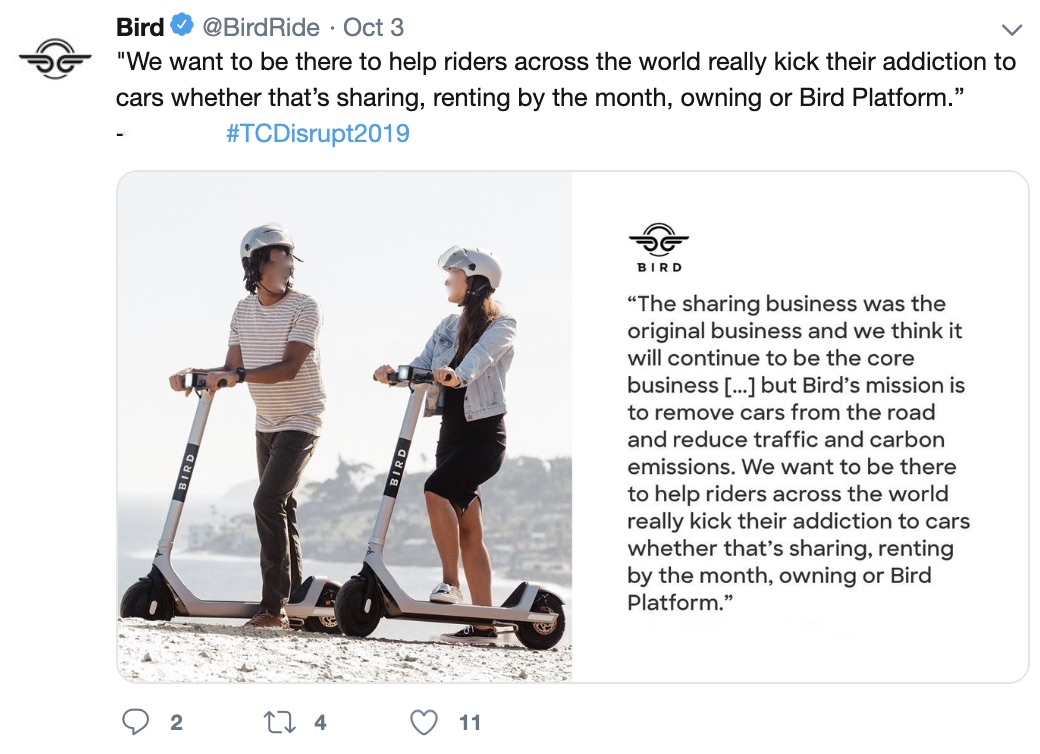 |
